# Fabrication of TiO_2_-Nanotube-Array-Based Supercapacitors

**DOI:** 10.3390/mi10110742

**Published:** 2019-10-31

**Authors:** Faheem Ahmed, Syed A. Pervez, Abdullah Aljaafari, Adil Alshoaibi, Hatem Abuhimd, JooHyeon Oh, Bon Heun Koo

**Affiliations:** 1 Department of Physics, College of Science, King Faisal University, P.O. Box-400, Al-Ahsa 31982, Saudi Arabia; aaljaafari@kfu.edu.sa; 2 Helmholtz Institute Ulm, Electrochemical Energy Storage, 89081 Ulm, Germany; syedatif.pervez@partner.kit.edu; 3 National Nanotechnology Center, King Abdulaziz City for Science and Technology, P.O Box 6086, Riyadh 11442, Saudi Arabia; habuhimd@kacst.edu.sa; 4 School of Materials Science & Engineering, Changwon National University, Changwon 51140, Korea; rnrghk9723@naver.com

**Keywords:** TiO_2_, nanotubes, supercapacitors, transmission electron microscopy (TEM), electrochemical

## Abstract

In this work, a simple and cost-effective electrochemical anodization technique was adopted to rapidly grow TiO_2_ nanotube arrays on a Ti current collector and to utilize the synthesized materials as potential electrodes for supercapacitors. To accelerate the growth of the TiO_2_ nanotube arrays, lactic acid was used as an electrolyte additive. The as-prepared TiO_2_ nanotube arrays with a high aspect ratio were strongly adhered to the Ti substrate. X-ray diffraction (XRD) and transmission electron microscopy (TEM) results confirmed that the TiO_2_ nanotube arrays were crystallized in the anatase phase. TEM images confirmed the nanotublar-like morphology of the TiO_2_ nanotubes, which had a tube length and a diameter of ~16 and ~80 nm, respectively. The electrochemical performance of the TiO_2_ nanotube array electrodes was evaluated using the cyclic voltammetry (CV), electrochemical impedance spectroscopy (EIS), and galvanostatic charge/discharge (GCD) measurements. Excellent electrochemical response was observed for the electrodes based on the TiO_2_ nanotube arrays, as the cells delivered a high specific capacitance of 5.12 mF/cm^2^ at a scan rate of 100 mV/s and a current density of 100 µA/cm^2^. The initial capacity was maintained for more than 250 cycles. Further, a remarkable rate capability response was observed, as the cell retained 88% of the initial areal capacitance when the scan rate was increased from 10 to 500 mV/s. The results suggest the suitability of TiO_2_ nanotube arrays as electrode materials for commercial supercapacitor applications.

## 1. Introduction

In recent years, because of their special properties including high power density, long cycle life, small size, cost effective, and good reversibility [1,2,3], supercapacitors have become one of the most important and promising energy storage devices. Supercapacitors have been used for various applications, such as portable electronic devices, pacemakers, hybrid electric vehicles, energy management and memory back-up systems [4,5,6]. For electrode materials, numerous transition metal oxides and conducting polymers have been used [7,8,9]. Among them, TiO_2_ has been recognized as one of the most capable candidates due to its inexpensive, naturally abundant, and environmentally safe (green) properties. Nowadays, most research focuses on enhancing the electrochemical capacitance and stability of the electrodes. The overall electrochemical performance of a supercapacitor is governed by the electrode materials, and electrodes with ordered structural morphologies could potentially deliver high capacitance. Thus, the key solution is the development of nanostructured electrodes containing enormously large surface area. A large surface area and small diffusion paths are required for electrons and ions [10,11,12,13,14,15] to accomplish fast redox reactions. To achieve this target, one-dimensional (1D) nanostructures such as nanotubes, nanowires, nanosheets, and mesoporous with controlled size, shape, crystallinity, and chemical composition could be considered. Thus, much work has been done to prepare 1D TiO_2_ morphology.

In particular, because of high surface area, good chemical stability, and wide potential window, 1D TiO_2_ nanotube arrays have largely been used as suitable electrode material for supercapacitors. Moreover, vertically aligned TiO_2_ nanotube arrays can be directly used as a supercapacitor electrode, as it gives a straight pathway for electron transport along the elongated axis of nanotubes to the Ti foil substrate. However, it is very difficult to synthesize TiO_2_ nanotubes that have a high aspect ratio [16]. During the charge/discharge process, a loosely adhered layer to the current collector side might be risky and become detached. Various groups have produced TiO_2_ nanotubes by different methods and successfully used them for supercapacitor electrodes [17,18,19,20].

In this work, we synthesized vertically aligned TiO_2_ nanotube arrays as an electrode material prepared using an electrochemical anodization method with a fast growth rate, high aspect ratio, and strong adherence to the metal surface for supercapacitor applications. The TiO_2_ nanotube arrays were grown vertically aligned with the Ti metal (current collector) and was strongly attached to the metal surface; thus, a binder was not required. The areal capacity of the as-prepared electrodes might be enhanced by growing high aspect ratio nanotubes. For a TiO_2_ nanotube layer thickness of ~16 μm, we have revealed stable supercapacitor performance for 250 charge/discharge cycles. Furthermore, at a higher current density of 100 µA/cm^2^ and a scan rate of 100 mV/s, a high specific capacitance of 5.12 mF/cm^2^ was observed. Structural and morphological information was obtained by X-ray diffraction (XRD), field emission scanning electron microscopy (FE-SEM), and transmission electron microscopy (TEM). The electrochemical performance of the as-prepared electrode was also studied using cyclic voltammetry (CV), electrochemical impedance spectroscopy (EIS), and the galvanostatic charge/discharge (GCD) method.

## 2. Experimental Details

All chemicals were used without any further purification upon received. Precleaned Ti foils (0.1 mm thick, 99.99% purity, Nilaco, Tokyo, Japan) were electrochemically anodized in an electrolyte composed of a suitable amount of lactic acid (1–1.5 M), 0.1 M ammonium fluoride, and the desired wt % of deionized (DI) water in ethylene glycol [21]. The anodization was conducted using a high-voltage potentiostat (OPS-22101, ODA, Seoul, Korea) at a DC voltage of 120 V for 300–500 s and an electrolyte temperature of 50–60 °C. The obtained samples were rinsed in ethanol and dried in an oven. For the crystalline phase of TiO_2_, samples were annealed at 500 °C for 1 h in air.

The crystallographic structure of the films was analyzed by an X’pert MPD 3040 X-ray powder diffractometer (PANalytical, Almerlot, Netherlands) operated at 40 kV and 30 mA with CuKα (1.541 Å) radiation within the scan range of 20°–80°. The morphology and the thickness of the film was characterized by FE-SEM using an FEI Quanta-250 FEG microscope (Thermo Scientific, Hillsboro, OR, USA). TEM micrographs and high-resolution (HR) images were obtained using a FE-TEM (JEOL/JEM-2100F version; Akishima, Tokyo, Japan) operated at 200 kV.

A two-electrode system was used to fabricate supercapacitors with TiO_2_ nanotube arrays as the active materials. The test coin cell consisted of a metal cap, a metal case with a polymer seal, a spring, two stainless-steel spacers, two current collectors coated with active materials, and a membrane separator. We used 1.2 M LiPF_6_ in EC/DMC (1:1, *v*/*v*) as an electrolyte. For the supercapacitors, coin cells were assembled in a vacuum glove box with argon atmosphere to avoid oxygen and moisture. The capacitive behavior of the supercapacitors was tested by CV, GCD, and EIS on an electrochemical workstation (GAMRY-3000, Warminster, PA, USA) at room temperature. Galvanostatic charging/discharging was performed at a current density of 100 µA/cm^2^ in a potential range of 0–2.8 V. CV was carried out at various scan rates in the range of 20–500 mV/s, while EIS analyses were performed by applying a perturbation voltage of 100 mV/s in a frequency range between 1 Hz and 100 kHz. The cycling stability of the supercapacitors was tested by using continuous charge/discharge cycling at a current density of 100 µA/cm^2^.

## 3. Results and Discussion

**Figure 1:**
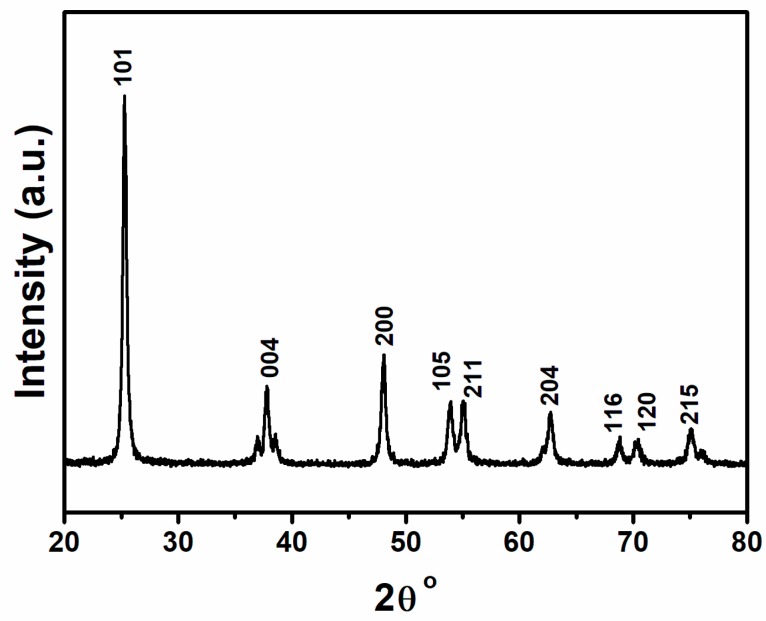
X-ray diffraction (XRD) pattern of TiO_2_ nanotube arrays prepared via the anodization method.

Figure 1 shows the XRD pattern of crystalline TiO_2_ nanotube arrays. It is clear from the pattern that the prepared TiO_2_ nanotube arrays showed characteristic diffraction peaks which were very well matched with standard pattern of JCPDS (01-084-1286) of anatase TiO_2_ crystals. No characteristic peaks of any impurities were detected, which demonstrates that the nanotubes had high phase purity, and the sharpness of the peaks indicates the high crystallinity of the TiO_2_ nanotube arrays.

The Ti foil was electrochemically anodized at 120 V and 60 °C for 500 s to obtain TiO_2_ nanotube arrays. The anodization conditions (voltage, temperature, and time) and the concentration of ammonium fluoride in the electrolyte along with DI water were optimized to achieve nanotube layers with the highest aspect ratio possible. The anodization process involved the formation and etching of TiO_2_ oxide layers. When equilibrium was reached between formation and etching, then porous oxide layers/arrays started to grow. In such a case, the metal oxides (MO_x_) formed were attacked by the fluorine (F^−^) ions originating from NH_4_F to form water-soluble metal fluoride species (MF_y_), resulting in nanotublar morphologies for the oxide layers. The reaction mechanism is shown below:

$${\mathrm{Ti}}^{4+}+6F^{-}\to {\left({\mathrm{TiF}}_{6}\right)}^{2-}$$

$${\mathrm{TiO}}_{2}+6F^{-}+\overset{H^{+}}{\to }{\left({\mathrm{TiF}}_{6}\right)}^{2-}+2H_{2}O$$

**Figure 2:**
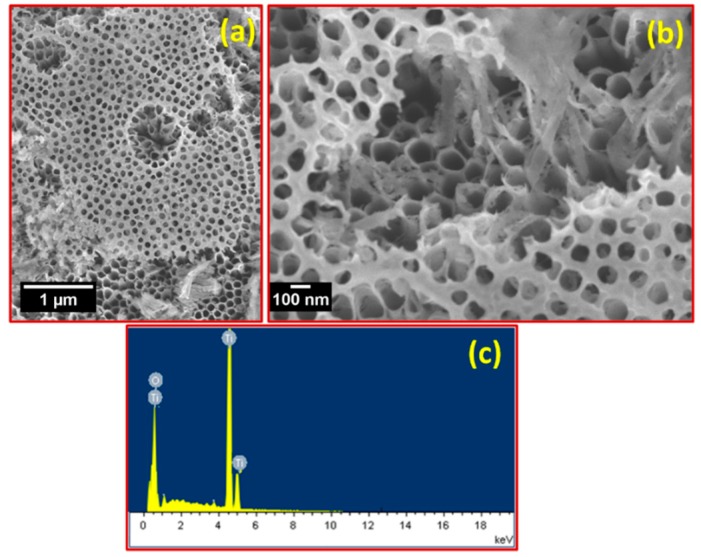
(**a**) Low and (**b**) high magnification field emission scanning electron microscopy (FE-SEM) images of TiO_2_ nanotube arrays. (**c**) Corresponding energy-dispersive X-ray spectroscopy (EDX) spectrum.

Figure 2a,b show the surface morphology of the respective TNTs with a layer thickness of ~16 μm. It is clear from the FE-SEM images that vertically aligned TiO_2_ nanotube arrays with tube openings ranging from ~80 to 100 nm were grown and that they possessed a high aspect ratio. These nanotubes grew in one direction and were open at the top (see Figure 2b). With the addition of lactic acid in the electrolyte, anodization could be performed at a higher voltage and no dielectric breakdown occurred [21]. The growth rate with lactic acid was significantly higher than that of the nanotubes grown in electrolytes without lactic acid [22,23]. The elemental composition of the TiO_2_ nanotube arrays was investigated using energy-dispersive X-ray spectroscopy (EDX). The EDX plot, as shown in Figure 2c, shows peaks of Ti and O for the nanotubes, which indicates that the TiO_2_ nanotube structures were composed of only Ti and O. No evidence of other impurities was found, which also confirms the high purity of the TiO_2_ nanotube arrays.

**Figure 3:**
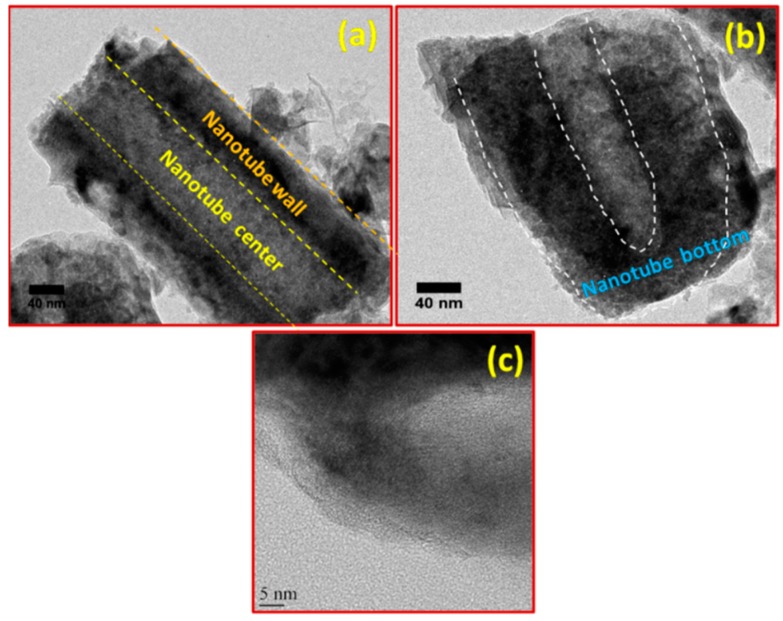
Transmission electron microscopy (TEM) images of TiO_2_ nanotubes: (**a**) middle portion, (**b**) bottom portion, and (**c**) corresponding high-resolution TEM (HRTEM) image.

Additional morphological characterization was accomplished using TEM, as shown in Figure 3a–c. Figure 3a shows a typical TEM image of the middle portion of the TiO_2_ nanotubes, while the bottom part of nanotubes is clearly shown in Figure 3b. It is worth noting from TEM images that the tubes were hollow and had a uniform thickness at the center, top, and bottom parts. The detailed atomic structure of the TiO_2_ nanotubes was characterized via HRTEM. The HRTEM image in Figure 2c shows that the ZnO nanotubes were highly crystalline and had lattice fringes corresponding to the anatase phase of TiO_2_.

**Figure 4:**
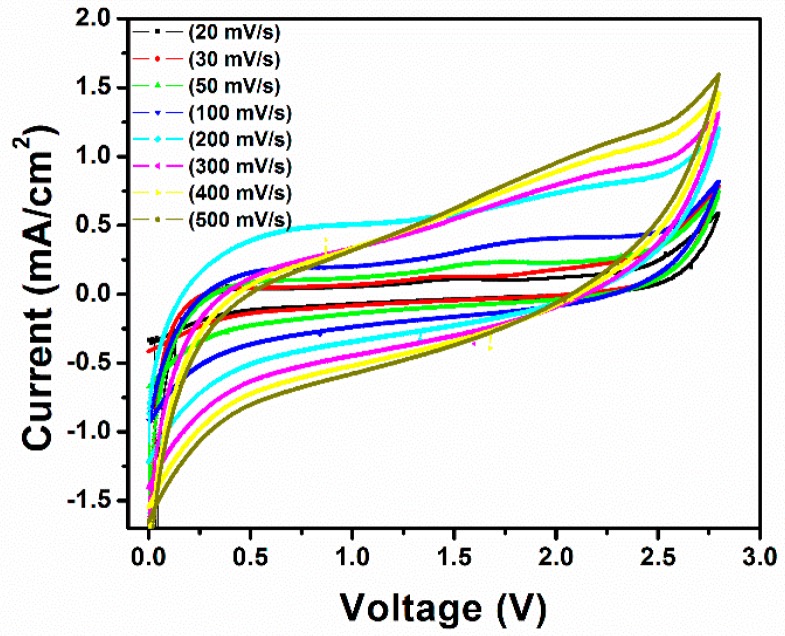
Cyclic voltammogram of TiO_2_ nanotube arrays at different scan rates, from 20 to 500 mV/s.

For energy storage applications, TiO_2_ nanotube arrays prepared by the anodization method were used as electrode materials for supercapacitors. Figure 4 shows the CV curve of TiO_2_ nanotube arrays at different scan rates of 20, 30, 50, 100, 200, 300, 400, and 500 mV/s in the potential window of 0–2.8 V. As shown in Figure 4, a quasi-rectangularly shaped curve can be clearly seen, which indicates ideal capacitive behavior. Additionally, as the scan rate increased, the current increased linearly; thus, the charge was primarily nonfaradic in nature. Capacitance performance of the TiO_2_ nanotube electrodes was further used for the detailed measurements. The specific capacitance (Cs) of the electrode at a scan rate of 100 mV/s and a current density of 100 µA/cm^2^ was 5.12 mF/cm^2^, which is almost 5 times higher than that of the TiO_2_/Ti electrode (11.70 F/g). In addition, our TiO_2_ nanotube arrays demonstrated excellent capacitance compared with other reported supercapacitors. Wang et al. [17] obtained specific capacitance of 17.7 F/g for TiO_2_-B nanotubes synthesized by a solvothermal method. Salari et al. [18] reported that a self-organized TiO_2_ nanotube array prepared by the anodic oxidation method had a specific capacitance of 2.6 mF/cm^2^ at 1 mV/s. However, these materials provide a non ideal response of specific capacitance and reversibility at higher current densities. In another report, Lu et al. [19] showed that the electrochemical performance of TiO_2_ could be improved by hydrogenation. They observed that TiO_2_ nanotube arrays hydrogenated at 400 °C attained the highest specific capacitance of 3.24 mF/cm^2^ at a scan rate of 100 mV/s and had excellent rate capability and long-term stability. In a study by Hui Wu et al. [20], TiO_2_ nanotube electrodes treated by electrochemical hydrogenation doping (TiO_2_-H) exhibited a specific capacitance of 5.42 mF/cm^2^ at a current density of 0.05 mA/cm^2^. Compared with the above-reported works, our TiO_2_ nanotube arrays showed excellent capacitance (5.12 mF/cm^2^) and reversibility behavior at a high current density of 100 µA/cm^2^ and a scan rate of 100 mV/s.

**Figure 5:**
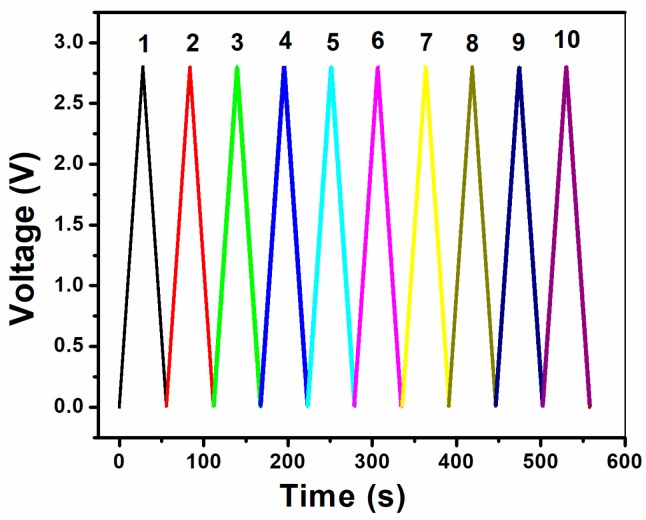
Galvanostatic charge/discharge curve of TiO_2_ nanotube arrays at 100 µA/cm^2^.

The charge/discharge performance of the TiO_2_ nanotube array electrodes was studied by the GCD method at a constant current density of 100 µA/cm^2^ in the potential range from 0 to 2.8 V. Figure 5 shows the typical GCD plot of the TiO_2_ nanotube array electrodes. It is clear from the figure that the whole curve is linear and symmetrical, which indicates the ideal capacitive characteristics and excellent electrochemical reversibility of the electrode.

**Figure 6:**
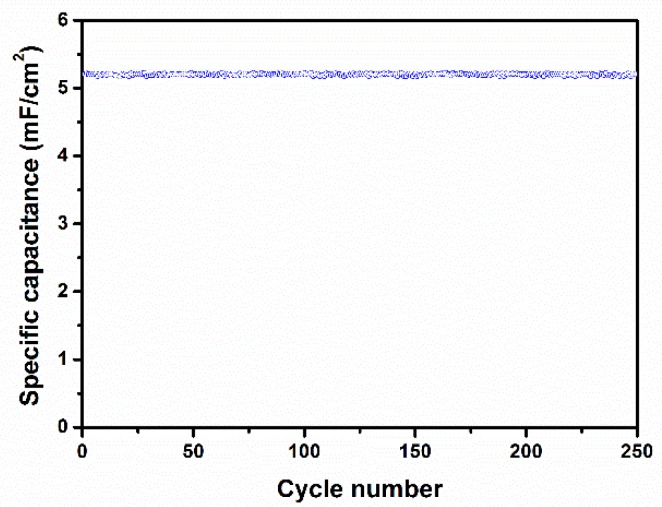
Variation of the specific capacitance of TiO_2_ nanotube array electrodes as a function of cycle number measured at 100 mV/s.

For the practical applications of supercapacitors, long-term cycle stability is an important parameter. Figure 6 shows the cycle stability of the TiO_2_ nanotube array electrodes examined by repeating the CV measurements between 0 and 2.8 V at a scan rate of 100 mV/s for 250 cycles. The electrode was found to exhibit an excellent long cycle life over all of the cycles. The specific capacitance as a function of cycle number was almost constant, even after 250 cycles, indicating the good cycling life of the electrode materials.

**Figure 7:**
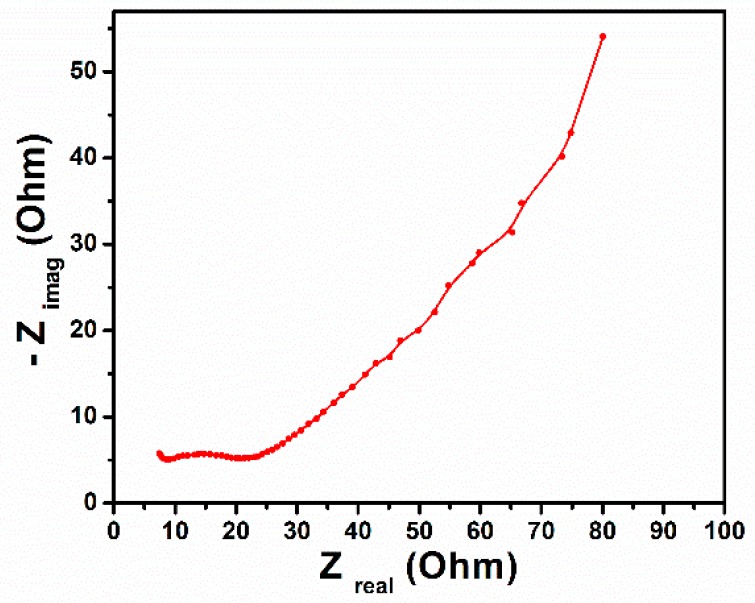
Electrochemical impedance spectra of TiO_2_ nanotube arrays from 100 kHz to 1 Hz in the form of Nyquist plots.

The EIS measurements were conducted at the open-circuit potential in the frequency range of 1–100 kHz with an AC perturbation of 100 mV. Figure 7 shows the Nyquist plot of the TiO_2_ nanotube arrays, the impedance plot shows a semicircle arc in the high-frequency, and a linear response in the low-frequency region [24]. The diameter of the semicircle corresponded to a resistance of 24 Ω, which indicates that the charge transfer resistance of the TiO_2_ nanotube arrays was low, further indicating that the charger transfer was much more conductive. The linear part at low-frequency region is related to the Warburg resistance (diffusive resistance) of the electrolyte into the electrode surface and diffusion/transport of an ion into the electrode surface. Moreover, a vertical line at imaginary part of impedance in low-frequency region, represents fast ion diffusion into the electrolyte and adsorption onto the electrode surface, showing an ideal capacitive behavior of electrodes [25].

## 4. Conclusions

In summary, TiO_2_ nanotube arrays with a high aspect ratio and a fast growth rate without any binder were successfully prepared by an electrochemical anodization technique and used as a supercapacitor electrode. XRD and TEM results confirmed the phase purity of the TiO_2_ nanotube arrays. TEM and SEM images confirmed the formation of uniform tubular structures with tube lengths and diameters of about ~16 and 80–100 nm, respectively, that were grown on the entire Ti surface. The electrochemical results showed that the prepared TiO_2_ nanotube array electrodes demonstrated ideal capacitive behavior and had a maximum specific capacitance of 5.12 mF/cm^2^ at a scan rate of 100 mV/s and a current density of 100 µA/cm^2^. In addition, the prepared electrode showed excellent reversibility with ca. 98% capacitance retention even after 250 cycles. These results reveal that the prepared TiO_2_ nanotube arrays are promising electrode materials for commercial supercapacitor applications. Supercapacitor devices based on the proposed electrode materials have the potential to replace the conventional thin-film Li-ion batteries for flexible electronic devices.

## References

[1] Kim D.W., Rhee K.Y., Park S.J. (2012). Synthesis of activated carbon nanotube/copper oxide composites and their electrochemical performance. J. Alloys Comp..

[2] Cheng J.P., Chen X., Wu J.S., Liu F., Zhang X.B., Dravid V.P. (2012). Porous cobalt oxides with tunable hierarchical morphologies for supercapacitor electrodes. Cryst. Eng. Comm..

[3] Wang Z., Maa C., Wang H., Liu Z., Hao Z. (2013). Facilely synthesized Fe_2_O_3_–graphene nanocomposite as novel electrode materials for supercapacitors with high performance. J. Alloys Comp..

[4] Lei Z., Christov N., Zhao X.S. (2011). Intercalation of mesoporous carbon spheres between reduced graphene oxide sheets for preparing high-rate supercapacitor electrodes. Energy Environ. Sci..

[5] Xia X., Hao Q., Lei W., Wang W., Sun D., Wang X. (2012). Nanostructured ternary composites of graphene/Fe_2_O_3_/polyaniline for high-performance supercapacitors. J. Mater. Chem..

[6] Liu W.W., Yan X.B., Lang J.W., Peng C., Xue Q. (2012). Flexible and conductive nanocomposite electrode based on graphene sheets and cotton cloth for supercapacitor. J. Mater. Chem..

[7] Yang X.H., Wang Y.G., Xiong H.M., Xia Y.Y. (2007). Interfacial synthesis of porous MnO_2_ and its application in electrochemical capacitor. Electrochim. Acta.

[8] Yang W., Gao Z., Wang J., Wang B., Liu Q., Li Z., Mann T., Yang P., Zhang M., Liu L. (2012). Synthesis of reduced graphene nanosheet/urchin-like manganese dioxide composite and high performance as supercapacitor electrode. Electrochim. Acta.

[9] Wang Y., Guo C.X., Liu J., Chen T., Yang H., Li C.M. (2011). CeO_2_ nanoparticles/graphene nanocomposite-based high performance supercapacitor. Dalton Trans..

[10] Lu X.H., Zheng D.Z., Zhai T., Liu Z.Q., Huang Y.Y., Xie S.L., Tong T.Y.X. (2011). Facile synthesis of large-area manganese oxide nanorod arrays as a high-performance electrochemical supercapacitor. Energy Environ. Sci..

[11] Xia X.H., Tu J.P., Mai Y.J., Wang X.L., Gu C.D., Zhao X.B. (2011). Self-supported hydrothermal synthesized hollow Co_3_O_4_ nanowire arrays with high supercapacitor capacitance. J. Mater. Chem..

[12] Feng D., Lv Y.Y., Wu Z.X., Dou Y.Q., Han L., Sun Z.K., Xia Y.Y., Zheng G.F., Zhao D.Y. (2011). Free-standing mesoporous carbon thin films with highly ordered pore architectures for nanodevices. J. Am. Chem. Soc..

[13] Jiang H., Yang L., Li C., Yan C., Lee P.S., Ma J. (2011). High–rate electrochemical capacitors from highly graphitic carbon–tipped manganese oxide/mesoporous carbon/manganese oxide hybrid nanowires. Energy Environ. Sci..

[14] Guo S.J., Dong S.J., Wang E.K. (2008). Constructing carbon-nanotube/metal hybrid nanostructures using homogeneous TiO_2_ as a spacer. Small.

[15] Jiang H., Zhao T., Ma J., Yan C.Y., Li C.Z. (2011). Ultrafine manganese dioxide nanowire network for high-performance supercapacitors. Chem. Commun..

[16] Ghicov A., Albu S.P., Hahn R., Kim D., Stergiopoulos T., Kunze J. (2009). TiO_2_ nanotubes in dye-sensitized solar cells: Critical factors for the conversion efficiency. Chem. Asian J..

[17] Wang G., Liu Z.Y., Wu J.N., Lu Q. (2012). Preparation and electrochemical capacitance behavior of TiO_2_-B nanotubes for hybrid supercapacitor. Mater. Lett..

[18] Salari M., Aboutalebi S.H., Chidembo A.T., Nevirkovets I.P., Konstantinov K., Li H.K.u. (2012). Enhancement of the electrochemical capacitance of TiO_2_ nanotube arrays through controlled phase transformation of anatase to rutile. Phys. Chem. Chem. Phys..

[19] Lu X.H., Wang G.M., Zhai T., Yu M.H., Gan J.Y., Tong Y.X., Li Y. (2012). Hydrogenated TiO_2_ nanotube arrays for supercapacitors. Nano Lett..

[20] Wu H., Li D., Zhu X., Yang C., Liu D., Chen X., Song Y., Lu L. (2014). High-performance and renewable supercapacitors based on TiO_2_ nanotube array electrodes treated by an electrochemical doping approach. Electrochim. Acta.

[21] So S., Lee K., Schmuki P. (2012). Ultrafast growth of highly ordered anodic TiO_2_ nanotubes in lactic acid electrolytes. J. Am. Chem. Soc..

[22] Ryu W.H., Nam D.H., Ko Y.S., Kim R.H., Kwon H.S. (2012). Electrochemical performance of a smooth and highly ordered TiO_2_ nanotube electrode for Li-ion batteries. Electrochim. Acta.

[23] Brumbarov J., Kunze-Liebhäuser J. (2014). Silicon on conductive self-organized TiO_2_ nanotubes–A high capacity anode material for Li-ion batteries. J. Power Sources.

[24] Liu K.K., Hu Z.L., Xue R., Zhang J.R., Zhu Z.J. (2008). Electropolymerization of high stable poly(3, 4-ethylenedioxythiophene) in ionic liquids and its potential applications in electrochemical capacitor. J. Power Sources.

[25] Salari M., Aboutalebi S.H., Konstantinov K., Liu H.K. (2011). A highly ordered titania nanotube array as a supercapacitor electrode. Phys. Chem. Chem. Phys..

